# Correlates of reward-predictive value in learning-related hippocampal neural activity

**DOI:** 10.1002/hipo.20535

**Published:** 2009-05

**Authors:** Murat Okatan

**Affiliations:** Laboratory of Cognitive Neurobiology, Department of Psychology, Boston UniversityBoston, Massachusetts

**Keywords:** reward pathway, action selection, prefrontal cortex, joint probability model, machine learning

## Abstract

Temporal difference learning (TD) is a popular algorithm in machine learning. Two learning signals that are derived from this algorithm, the predictive value and the prediction error, have been shown to explain changes in neural activity and behavior during learning across species. Here, the predictive value signal is used to explain the time course of learning-related changes in the activity of hippocampal neurons in monkeys performing an associative learning task. The TD algorithm serves as the centerpiece of a joint probability model for the learning-related neural activity and the behavioral responses recorded during the task. The neural component of the model consists of spiking neurons that compete and learn the reward-predictive value of task-relevant input signals. The predictive-value signaled by these neurons influences the behavioral response generated by a stochastic decision stage, which constitutes the behavioral component of the model. It is shown that the time course of the changes in neural activity and behavioral performance generated by the model exhibits key features of the experimental data. The results suggest that information about correct associations may be expressed in the hippocampus before it is detected in the behavior of a subject. In this way, the hippocampus may be among the earliest brain areas to express learning and drive the behavioral changes associated with learning. Correlates of reward-predictive value may be expressed in the hippocampus through rate remapping within spatial memory representations, they may represent reward-related aspects of a declarative or explicit relational memory representation of task contingencies, or they may correspond to reward-related components of episodic memory representations. These potential functions are discussed in connection with hippocampal cell assembly sequences and their reverse reactivation during the awake state. The results provide further support for the proposal that neural processes underlying learning may be implementing a temporal difference-like algorithm.

## INTRODUCTION

The temporal difference (TD) learning algorithm was introduced to address the temporal credit assignment problem whereby proper reward-predictive values are computed for each of a sequence of actions that ultimately result in success or failure (Sutton and Barto, 1981, 1987; Barto and Sutton, 1982; Sutton, 1988). It is widely used as a reinforcement learning algorithm in machine learning (Barto et al., 1983; Tesauro, 1994; Kaelbling et al., 1996; Singh and Bertsekas, 1997; Sutton and Barto, 1998). Two key signals of this algorithm are the predictive value (*V*(*t*)), and the prediction error (δ(*t*)) signals. Numerous studies of reinforcement learning in humans, nonhuman primates and bees have shown that these signals provide a compelling explanation of the patterns of neural activity and behavior that are observed during learning (Montague et al., 1995, 1996, 2004, 2006; Schultz et al., 1997; Suri and Schultz, 1999, 2001; Kakade and Dayan, 2000; Waelti et al., 2001; O'Doherty et al., 2003; McClure et al., 2004; Schultz, 2004; Seymour et al., 2004; Tanaka et al., 2004; Niv et al., 2005).

In monkeys that learn behavioral reactions, the response of midbrain dopamine neurons to reward is remarkably similar in its learning-dependent characteristics to the prediction error δ(*t*) of a TD learning algorithm that is trained on the same task (Montague et al., 1996, 2004; Schultz et al., 1997; Suri and Schultz, 1999; Kakade and Dayan, 2000; Waelti et al., 2001; Niv et al., 2005). Before learning the association between a stimulus and the reward that the stimulus reliably predicts, these neurons exhibit a phasic response to reward delivery. With learning, the timing of this response gradually shifts from the time of reward delivery to the time of stimulus presentation. After learning is established, the response occurs immediately following the stimulus that signals the future reward (Romo and Schultz, 1990; Ljungberg et al., 1992; Schultz et al., 1993). Correlates of δ(*t*) are found, during classical conditioning and higher-order learning in humans, in the activity of brain areas involved in reward processing, including the ventral tegmental area, substantia nigra, and ventral striatum (McClure et al., 2003, 2004; O'Doherty et al., 2003; Montague et al., 2004, 2006; Seymour et al., 2004; Tanaka et al., 2004). On the other hand, human fMRI studies of associative and higher-order learning revealed that correlates of *V*(*t*) are found in the ventral midbrain (O'Doherty et al., 2006), anterior insula cortex and the brainstem (Seymour et al., 2004), medial prefrontal cortex and bilateral insula, and temporal pole and the hippocampus (Tanaka et al., 2004). The blood-oxygen-level-dependent brain activity signal recorded in fMRI studies is thought to be correlated with postsynaptic events, and to reflect the neural processing occurring within a brain area (Attwell and Iadecola, 2002). This suggests that the δ(*t*) and the *V*(*t*) signals detected in the fMRI studies reflect functional processes expressed within the indicated anatomical locations. Direct measurements of neural spiking activity in electrophysiological studies in monkeys also found that cue-related activity of perirhinal neurons carries associative information about the temporal distance to future rewards, similar to *V*(*t*) (Liu and Richmond, 2000). These findings are consistent with the proposal that the medial temporal cortex may be involved in the representation of the predictive value signal *V*(*t*) (Schultz, 2000). Other electrophysiological evidence suggested that correlates of *V*(*t*) are also found in the dorsolateral prefrontal cortex and striatum in monkeys (Barraclough et al., 2004; Samejima et al., 2005).

Reward-related activity has been found in the spiking activity of rodent and primate hippocampal neurons during reinforcement learning experiments (Hölscher et al., 2003; Rolls and Xiang, 2005), and has also been suggested to be linked to a reinforcement learning mechanism similar to the TD learning algorithm (Foster and Wilson, 2006). Here, it is shown that the learning-related spiking activity of some hippocampal neurons (changing cells; Wirth et al., 2003) in monkeys performing an associative learning task (location-scene association task; Wirth et al., 2003) is correlated with the reward-predictive value signal *V*(*t*) generated by a TD learning algorithm that is trained on the same task. The time course of the activity of the changing cells exhibits key characteristics of *V*(*t*). Namely, both start changing first near the time of reward delivery and propagate backward in time with learning, toward stimuli that have reward-predictive value. The goal of the article is to provide answers to the following questions: Can the learning-related behavioral responses of a subject and the activity of the changing cells be explained in a framework that couples them through the TD learning algorithm? Is such an explanation compatible with other experimental data suggesting that the activity of some hippocampal neurons contains reward-related information? If the activity of some hippocampal neurons contains information about reward-predictive value, how may this information contribute to the function of the hippocampus according to current theories of hippocampal function?

## THEORY AND METHODS

### Theory of the TD Algorithm

The main goal of the TD algorithm is to compute a running estimate of the discounted sum of all future rewards in a trial (Schultz et al., 1997; Suri and Schultz, 2001; O'Doherty et al., 2003; Montague et al., 2004)


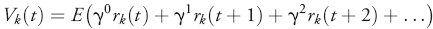
(1)

where *k* is the trial number, *r_k_*(*t*) is the reward at time *t* relative to trial onset, *E*(·) denotes the expected value of the sum of future rewards up to the end of the trial, and 0 ≤ γ ≤ 1 is a discount factor that attenuates the impact of late arriving rewards when γ < 1. This function is termed the predictive value function. An estimator of *V*(*t*) is often formulated as a linear combination of temporal basis functions *x^i^*(*t*) (Montague et al., 1996; Schultz et al., 1997; Suri and Schultz, 1999, 2001; O'Doherty et al., 2003), which may be interpreted as time-delayed versions of input signals whose reward-predictive value is learned by the algorithm (see Appendix, Eq. (A5))


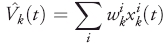
(2)

It is assumed that the compound signal 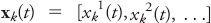 is Markov, such that the probability structure of the reward at trial *k* is completely specified knowing only **x**_*k*_(*t*) (Sutton and Barto, 1998). The TD algorithm computes the weights of these basis functions iteratively according to the weight update rule (Schultz et al., 1997; O'Doherty et al., 2003; Montague et al., 2004)


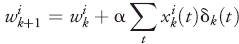
(3)

where α is the learning rate of the system, and δ(*t*) is an error signal that measures the difference between the predicted and the observed reward at time *t*


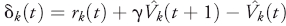
(4)

It serves as a teaching signal that controls how the associative weights coupling the input signal to its predictive value are modified. In the learning task considered here the reward is all or none (1 or 0) and is only available at the end of the trial. The weights and the value function vary between 0 and 1 under these conditions if α, γ, and the initial weights are between 0 and 1 (see Appendix).

### The Associative Learning Task

**FIGURE 1:**
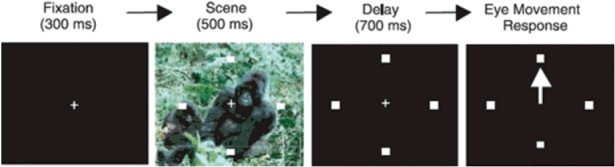
The display sequence in a typical trial of the location-scene association task. Reprinted with permission from Wirth et al., Science, 2003, 300, 1578–1581, ©American Association for the Advancement of Science.

The location-scene association task consists of learning arbitrary associations between complex visual scenes and superimposed target locations (Fig. 1). Each trial of the task starts with a black screen and a central fixation point. The subjects (two adult rhesus monkeys (*Macaca mulatta*)) are expected to maintain their gaze at this point until the end of the trial. The black screen is presented for 300 ms. Then, a complex visual scene is presented for 500 ms. Superimposed on this scene are four small white squares indicating target locations. After the scene stimulus is turned off, the target locations remain on the screen for a delay period of 700 ms. At the end of this period, the fixation point disappears, signaling to the subject that it is time to make an eye movement to one of the four target locations. Each scene is correctly associated with only one target location. The subject is rewarded if the correct location is selected. The monkeys were presented with two to four novel scene stimuli in each session in a pseudorandom order.

Wirth et al. recorded from 145 cells throughout the hippocampal region (Dentate Gyrus, CA3, CA1, Subicular Complex), of which 89 exhibited scene-selective activity during the scene or the delay period of the task and, of these, 69 showed significant activity relative to baseline (fixation period activity). The scene and/or the delay period activity of 25 of these 69 cells was significantly correlated with the behavioral learning curve (a total of 37 scene and delay cases). These cells were called the “changing cells” (Wirth et al., 2003). Two types of changing cells were identified. Sustained-changing cells (14/25) increased (12/14) or decreased (2/14) their activity relative to baseline with learning, and baseline-sustained changing cells (11/25) initially responded with increased (3/11) or decreased (8/11) activity, and returned to the baseline level with learning (Wirth et al., 2003).

### Parameter Estimation

In this section, the parameters of the TD algorithm are estimated for individual neuron models. The next section explains how these estimates are used in neurons that are coupled within a network. Each trial of the location-scene association task is modeled as a process with 15 equal time steps, where each time step lasts 100 ms as in previous studies (Montague et al., 1996; Schultz et al., 1997; Suri and Schultz, 1999, 2001). The time steps 1–5, 6–12, 13–15 correspond to the scene, delay, and response periods of the task, respectively, matching the relative timing of these periods in the actual location- scene association experiment (Wirth et al., 2003).

The model may start in a naïve state where the initial weights are zero (Schultz et al., 1997), or it may start with a previously acquired memory trace, modeled here by setting the initial weights to one. The input signal 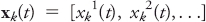 is a series of time-delayed pulses that is triggered at scene onset, which is usually referred to as a complete serial compound or tapped-delay line (see Appendix, Eq. (A5); Schultz et al., 1997).

**FIGURE 2:**
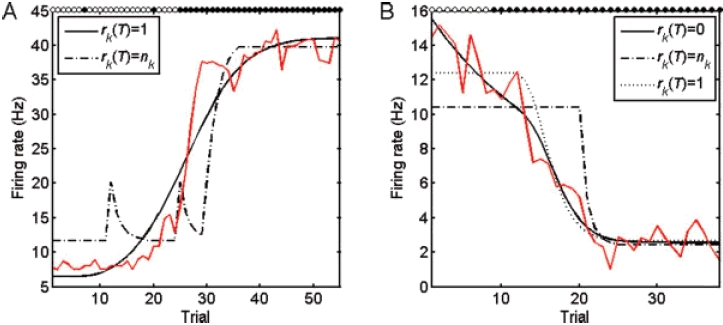
Learning-related hippocampal neural activity and reward-predictive value. The average firing rates (red) of a sustained-changing cell (A) and a baseline-sustained changing cell (B) relative to baseline. The legend shows the reward-schedules used in fitting the data. Maximum likelihood estimates for the best fits (solid black curves): (A) 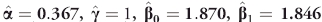; (B) 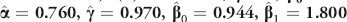. The white and the black dots at the top of each graph indicate the incorrect and the correct responses of the subject, respectively. The estimated learning and neural change trials for these cases were 25 and 26 (A), 9 and 19 (B), respectively (Wirth et al., 2003). The firing rate increases in (A) (*P* = 0.01) and decreases in (B) (*P* = 0.03) before the estimated learning trial according to the slope test (see section “Slope of neural change before learning” for Methods). Data extracted from Figure 2 of Wirth et al., Science, 2003, 300, 1578–1581, © American Association for the Advancement of Science.

For a given pair of α and γ, Eqs. (2)–(4) are iterated using one of the following three reward schedules: *r_k_*(*T*) = *n_k_*, *r_k_*(*T*) = 1, or *r_k_*(*T*) = 0, where the time *T* indicates the end of the trial and *n_k_* is the outcome of a subject's behavioral response such that *n_k_* = 1 if the response is correct, and *n_k_* = 0 otherwise, as shown in Figure 2. The reward signal is zero at all other times.

The average reward-predictive value generated by the TD algorithm is computed in the scene and the delay periods of the task, and the firing rates observed in Figure 2 are fit as a function of this signal using a generalized linear model (GLM) with Poisson distribution and logarithmic link function (McCullagh and Nelder, 1989), such that the expected value of the average firing rate in a task period at trial *k* is exp(
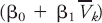
), where 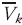 is the average reward-predictive value in the scene or the delay period of the task. The logarithmic link function ensures the non-negativity of the firing rate. The parameter α is varied in the range [0,1] in 80 linear steps and γ is varied in the range [0,1] in 100 linear steps and the maximum likelihood estimates of these parameters are determined in these ranges.

The relation between the behavioral responses and the neural activity is modeled using a GLM with Binomial distribution and logit link function such that the behavioral response *n_k_* at trial *k* is a Bernoulli random variable with probability


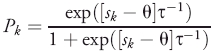
(5)

where *s_k_* is the average firing rate of the neuron in the scene or the delay period of the task. The maximum likelihood estimates of the parameters θ and τ are determined from Figure 2.

### A Model of Competing Changing Cells

To show that the time course of the learning-related neural activity and behavior observed during the location-scene association task may be explained using the TD learning algorithm, a network of simulated cells are trained on the task, using the parameters estimated from Figure 2.

In the simulated location-scene association experiment, the task is to associate four different scenes (*A, B, C, D*) with four different locations (*a, b, c, d*). A simulated cell is assigned for each of the 16 possible location-scene pairs as in other models where one TD model is assigned to each event in multievent tasks (Suri and Schultz, 2001). Four of the cells correspond to rewarded (correct) location-scene pairs. These cells are referred to here as the correct cells. The remaining 12 cells are referred to as the incorrect cells. The full model is a joint probability model for the learning-related ensemble spiking activity of the cells and the behavioral responses of the model across the entire session (see Appendix).

#### Spiking activity in the simulated neurons

The spiking activity of a cell is controlled by the value function that measures the reward-predictive value of the cell's location-scene pair


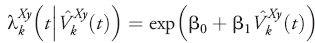
(6)

where 
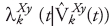
 is the conditional intensity function of the cell *C^Xy^* corresponding to the scene *X* and location *y*. The conditional intensity function is used to compute the probability of a spike in the brief interval (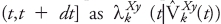) *dt* (Daley and Vere-Jones, 2003). Using *dt* = 1 ms, a spike is generated in a given time bin with this probability.

#### Behavioral responses of the network

At each trial, each scene has equal probability of being presented. A trial begins with the presentation of a scene (e.g., *A*), which activates the cells that receive input from that scene (e.g., *C^Aa^, C^Ab^, C^Ac^, C^Ad^*). The coupling between neural activity and behavioral response is modeled as a two-stage process. In the first stage, the neurons activated by the presented scene compete among themselves (Cahusac et al., 1993; Suri and Schultz, 1999). The cell that has the highest firing rate in the delay period wins the competition. If there is a tie, then one of the candidates is selected with equal probability. Then, at the final decision stage, a location *y* is selected as the response, with a probability that depends on the delay period firing rate 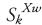 of the winner cell *C^Xw^*.


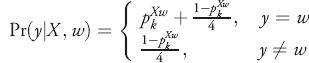
(7)


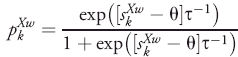
(8)

According to this rule, if the decision mechanism fails to choose the location associated with the winner cell with probability 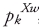, then a location is selected with equal probability among the four possibilities. Action selection is performed using the delay period activity because the values that were used for the parameters θ and τ in this simulation were estimated using Eq. (5) with the behavioral responses and the delay-period activity shown in Figure 2A: 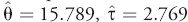. The design of this scheme was motivated by the assumption that a population of hippocampal neurons wins a competition and the activity of the winning population contains information about the response probability at each trial. This relation between the neural activity and the response probability is captured by the binomial regression in Eqs. (5) and (8).

#### Learning in the model

The model neurons may start in a naïve state, where the initial weights are 0, or they may start with a previously acquired reward-predictive value, modeled here by setting the initial weights of a neuron to 1. At the end of each trial, the input weights of the winner cell *C^Xw^* are updated according to the TD algorithm (Eqs. (3) and (4)) using a reward of 1 (0) if the model's choice is correct (incorrect). The input weights of the cells associated with the same scene but different locations are updated using the opposite reward. Namely, if the choice is correct, then they receive no reward (0), otherwise they each receive a reward of 1. The input weights of the cells associated with the other scenes remain unchanged at that trial. Therefore, in this scheme, a winner cell that corresponds to an incorrect location-scene pair may nonetheless get reward if the decision stage chooses the correct location by chance. Reciprocally, a winner cell that corresponds to a correct location-scene pair may fail to receive reward if the decision stage chooses a wrong location by chance. On the other hand, regardless of whether they correspond to correct or incorrect location-scene associations, cells that lose the competition at a given trial receive or do not receive reward, depending on whether the behavioral response at that trial is incorrect or correct, respectively.

### Neural Change and Behavioral Learning Trials in the Network

In a typical behavioral neurophysiology experiment, the state of the neural system is observed at the level of spike trains of individual neurons. Therefore, experimental studies have defined the neural change trial as the trial at which a significant change is detected in the spiking activity of a neuron during some task period (Cahusac et al., 1993; Wirth et al., 2003). This trial is necessarily later than the trial at which smaller but potentially significant changes may occur in the neural substrate, such as synaptic changes. Such smaller changes in neural substrate may manifest themselves as slight changes in firing rate that may not reach statistical significance when analyzed between pairs of trials, but may be consistent trends, such as consistent increases or decreases in firing rate as a function of trial number. Such consistent trends may be detected by fitting neural activity as a function of trial number and testing the slope of the fit for significance. This is explained in section “Slope of neural change before learning.”

The neural change trial may be defined according to multiple different criteria. For illustration purposes, it is defined here as the earliest trial *k* at which the difference between the firing rates at trial *k* and trial 1 significantly differs from zero and remains different for the rest of the session. This is determined by computing the 95% confidence interval of this difference using 1,000 independent simulated learning sessions and identifying the earliest trial at which this confidence interval does not contain zero for the rest of the session.

The behavioral learning trial is determined based on the probability of making a correct response by chance, which is determined by the number of potential actions available to the subject. In the location-scene association task, this probability is 0.25. Equations (7) and (8) provide the network's probability of correct response at each trial given the firing rates of all neurons. In the remainder of this article, this probability is referred to as the probability of correct response given the ensemble spiking activity. It has a distribution that depends on the firing rate distributions of the neurons. This distribution is computed by generating 100,000 simulated firing rates at each trial given the neural activity and the behavioral responses generated in the previous trials. The trial at which this probability exceeds the chance level with 95% confidence and remains above that level for the rest of the session is taken as the behavioral learning trial of the network.

For comparison, the neural change and the behavioral learning trials are estimated using the methods of Wirth et al. (2003), as explained next.

#### Behavioral learning trial estimate

The trial at which behavioral learning occurs is estimated using the state-space model of learning (Smith and Brown, 2003; Smith et al., 2004) as described in Wirth et al. (2003). In this model, behavioral responses are Bernoulli random variables (i.e., 0 or 1) with a probability that depends on a latent state process representing the level of learning. The state process is modeled as a Gaussian random walk. Given the binary behavioral responses of the learning agent, the method estimates the probability of making a correct response at each trial using the Expectation-Maximization algorithm (Dempster et al., 1977). It determines the learning trial as the trial at which this estimate exceeds the chance level (0.25) with 95% confidence and remains above that level for the rest of the experiment. This learning trial is termed the ideal observer learning trial with level of certainty 0.95 [IO (0.95)] (Smith et al., 2004). This nomenclature is due to the estimation of the learning state variable from the perspective of an ideal observer: the value of the learning state process at each trial is estimated after seeing the outcomes of all of the trials in the experiment (Smith et al., 2004).

**FIGURE 4:**
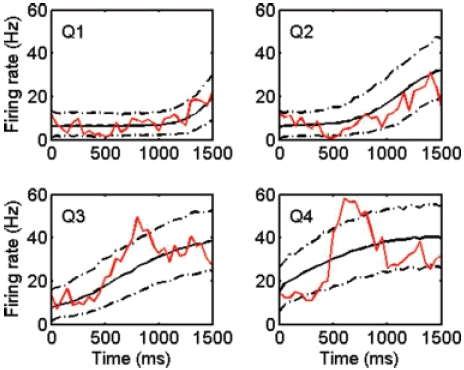
Neural activity in different learning quartiles. The panels show the firing rate estimates of a real changing cell (red) (from Fig. 3A of Wirth et al., 2003) and the firing rate estimates of neuron 1 obtained in 1,000 simulations using the same analysis. The solid black curve shows the average firing rate estimate. The dashed curves show the 95% confidence interval of the model's estimated firing rate. Learning was detected in all 1,000 simulations. Data extracted from Figure 3 of Wirth et al., Science, 2003, 300, 1578–1581, © American Association for the Advancement of Science.

**FIGURE 6:**
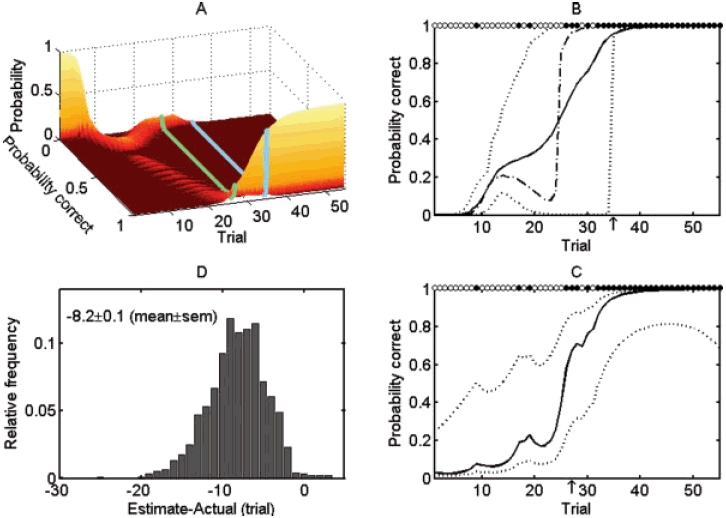
The network's decision making. (A) The probability density function of the model's probability of correct response given the ensemble spiking activity at each trial during a simulated learning session. The superimposed lines indicate the estimated (green, 27) and the actual (blue, 35) learning trials of the network in this session. (B) The mean (solid line) and the median (dashed line) of the distribution in (A). Dotted lines indicate the 5th and the 95th percentiles of the distribution. The 5th percentile exceeds the chance level at trial 35 (learning trial, arrow). The mean (solid line) corresponds to the probability of correct response given the value functions of all cells. (C) The maximum likelihood estimate (solid line) of the probability of correct response, and its 90%; confidence interval (dotted lines) computed using the state-space model of learning (Wirth et al., 2003; Smith et al., 2004). The lower confidence bound exceeds the chance level at trial 27 (arrow). (B, C) The white (black) dots indicate the incorrect (correct) responses of the network. (D) The distribution of the difference between the estimated and the actual learning trials in 963/1,000 simulations. The average difference is −8.2 ± 0.1 (s.e.m) trials. The network did not learn the association in one of the sessions. The state-space model of learning did not detect learning in 37 sessions. All results were obtained using the parameters in Figure 3.

This analysis is performed using the software available at the link provided by Smith et al. (2004), https://neurostat.mgh.harvard.edu/BehavioralLearning/Matlabcode, using the parameters specified in Wirth et al. (2003) (The startflag parameter was 0 in the analysis shown in Fig. 4, as in Wirth et al. (2003), and was 2 for all other analyses. This parameter takes on the values 0, 1, and 2, and controls the initial condition of the latent state process of the model. When startflag is zero, the initial probability of correct response is fixed at the chance level (0.25). When it is 1, the initial value is estimated from the data, and when it is 2, the model is not constrained by the initial value of the latent state process. The latter condition resulted in the smallest average difference between the estimated and the actual learning trials in Fig. 6).

#### Neural change trial estimated using raw firing rates

The neural change trial is estimated using the change point test for continuous variables (Siegel and Castellan, 1988). At each trial, the average firing rate in a task period is computed from the raw spike count observed in that period. The change point test is then used to determine the trial at which a significant change is detected in the firing rate. Wirth et al. (2003) noted that estimating the firing rate from raw spike counts yields noisy estimates of the change trial. Accordingly, they used the change point test on firing rate estimates that were obtained using adaptive filtering, as explained next.

#### Neural change trial estimated using adaptive filtering

Adaptive filtering is a parameter estimation method in which the estimates are updated in real time as new data become available (Haykin, 1996; Brown et al., 2001). It allows computing instantaneous estimates of dynamic parameters as a function of previous estimates and new data observations. Here, this method is implemented as described by Wirth et al. (2003) to filter the simulated spike trains generated by the model in order to estimate the firing rate underlying the spiking activity. Then, at each trial, the average firing rate is computed in different task periods using the adaptive filter estimate. The change point test for continuous variables (Siegel and Castellan, 1988) is used to determine the trial at which a significant change is observed in the firing rate (Wirth et al., 2003).

### Slope of Neural Change Before Learning

**FIGURE 7:**
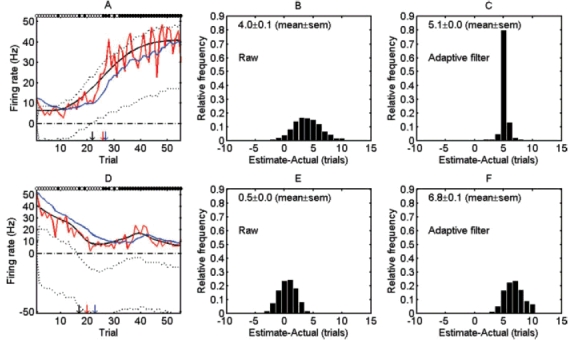
Neural change trials of the simulated neurons. (A, D) The average firing rates (red) of neuron 1 in the delay period of the task (A) and neuron 2 in the scene period of the task (D), observed during the session illustrated in Figure 6. The plots show the firing rate estimated using adaptive filtering (blue), the expected firing rate in the same task period computed using the conditional intensity function (solid black), and the 95% confidence interval of the difference between the firing rate at each trial and the firing rate at the first trial, obtained in 1,000 simulated learning sessions (dotted lines). The dashed horizontal line indicates the level zero for this difference. The lower (upper) dotted line crosses this level at trial 22 in (A) (17 in (D)) (black arrow), which defines the neural change trial for these neurons. The neural change trial estimates obtained using the change point test on the raw firing rates and on the firing rates estimated using adaptive filtering are shown by the red and blue arrows, respectively. In (A) they are 26 and 27, in (D) they are 20 and 23, respectively. The white and the black dots at the top indicate the incorrect and the correct responses of the network, respectively. (B, C, E, F) The distribution of the difference between the estimated and the actual neural change trials in 1,000 simulations. The average difference for each method is indicated in the graphs. The estimated behavioral learning trial is 27 from Figure 6. The neural activity exhibits a significant trend of change before the estimated learning trial according to the slope test (*P* = 0.014 in A, *P* = 0 in D; see section “Slope of neural change before learning” for Methods).

To determine whether the firing rate of a neuron exhibits a significant trend of change before the estimated learning trial, Poisson regression fits of second (Figs. 2A, 7A) and first (Figs. 2B, 7D) order polynomials are fit to the data as a function of trial number at trials before the estimated learning trial. The statistical significance of the quadratic (Figs. 2A, 7A) and the linear (Figs. 2B, 7D) slopes is determined using the bootstrap method (Efron and Tibshirani, 1998) as follows. First, the slope is obtained for the observed firing rates at trials preceding the estimated learning trial. Next, 10^4^ bootstrap replicates of the slope parameter are computed using random permutations of the observed rates at those trials. Finally, the *P*-value of the observed slope parameter is obtained as the fraction of replicates that are more extreme than or equal to the observed slope parameter.

### Software

All analyses were conducted using custom software written in Matlab (MathWorks, Natick, MA), except for the estimation of the behavioral learning trial, as explained in section 7 “Behavioral learning trial estimate.”

## RESULTS

### Parameter Estimation

Figure 2A shows the average firing rate of a sustained-changing cell in the delay period of the task and the outcome of the behavioral responses of the subject (*n_k_* = 1: correct, *n_k_* = 0: incorrect) at trials where a particular scene was shown during the performance of the task (Wirth et al., 2003). The neural activity is fit as a function of the reward-predictive value using the reward schedules *r_k_*(*T*) = *n_k_* and *r_k_*(*T*) = 1. In both cases, the initial weights of the TD algorithm are zero. In the first reward schedule, the fit lags the actual neural activity by a wide margin. This is expected because the reward-predictive value increases only after the algorithm receives reward, which occurs only at correct trials. Thus, the increase in reward-predictive value occurs only after the subject starts making correct responses. This suggests that a better fit to the data would be obtained under this model if the algorithm somehow received reward at least at some of the incorrect trials before behavioral learning. This idea is illustrated by the solid black curve in Figure 2A, which is obtained by fitting the model using the second reward schedule. This fit is significantly better than the previous according to a likelihood ratio test (log likelihood ratio: 103.6, *P* = 0, under the χ^2^ distribution with 1° of freedom).

Figure 2B shows the average firing rate of a baseline-sustained changing cell in the scene period of the task and the behavioral responses of the subject at trials where a particular scene was shown during the performance of the task (Wirth et al., 2003). Here the firing rate is fit using the reward schedules *r_k_*(*T*) = *n_k_*, *r_k_*(*T*) = 1, and *r_k_*(*T*) = 0. In the first two reward schedules, the initial weights of the algorithm are zero, whereas in the third, they are one. The latter condition may be viewed as the extinction of a previous memory trace. This reward schedule fits the data significantly better than the first reward schedule (log likelihood ratio: 17.2, *P* = 3.5 × 10^−5^) and almost significantly better than the second reward schedule (log likelihood ratio: 3.5, *P* = 0.06). Because the firing rate decreases significantly before the behavioral learning (Fig. 2B; *P* = 0.03; see section “Slope of neural change before learning”), and because this decrease is only captured by the model in the third reward schedule, it is proposed that the neural data in Figure 2B are best fit by the model under this reward schedule.

### Reward Inversion Through Competitive Interactions

The best fits to the data in Figure 2 are obtained using positive reward even at incorrect trials (A) or no reward even at correct trials (B). Such an inversion of the reward signal might occur within a competitive network of neurons where the reward signal is gated by competitive interactions. This competition is implemented as described in section “Behavioral responses of the network.”

To illustrate how neural activity of the type illustrated in Figures 2A and 2B may be generated within a network of competing neurons, neurons of two different types are included in the competitive network model. For simplicity, the activity of a group of four neurons that respond to a particular scene is illustrated. All model parameters are estimated from the data in Figure 2A except for the learning rate α and the discount rate γ for neuron 2, which are estimated from the data in Figure 2B. The initial weights are one for neuron 2, and zero for the other neurons. Neuron 1 is designated as a correct neuron, whereas the other neurons are incorrect neurons.

### Changes in the Predictive-Value Function During Learning

**FIGURE 3:**
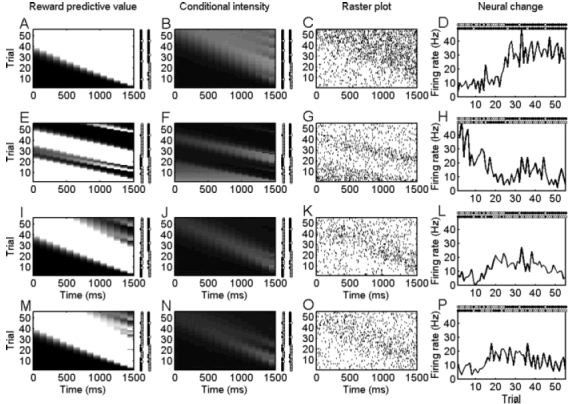
Learning-related changes in the reward-predictive value function and dependent processes. (A, E, I, M) The value function starts changing first near the end of the trial and then the change propagates backward toward the scene period as learning progresses. (B, F, J, N) The conditional intensity functions for the same simulated neurons. The intensity increases with increasing whiteness for the value function and the conditional intensity function. (C, G, K, O) The raster plots of the simulated spiking activity driven by the conditional intensity functions. (D, H, L, P) The average firing rates in the scene (H) and the delay (D, L, P) periods of the task computed from raw spike counts. White (black) dots on the right in vertically arranged sequences, and at the top in horizontally arranged sequences indicate the incorrect (correct) responses of the model. The left and the bottom dot sequences show the reward signal received by each cell (white: 0; black: 1).

The simulated behavior of the network is shown in Figure 3. In a typical experiment, the predictive value function associated with a location-scene pair exhibits learning-dependent changes as the TD algorithm assigned to that function receives feedback regarding the response given at each trial (Figs. 3A,E,I,M). The value function is zero in the first trial for neurons 1, 3, and 4, and it is one for neuron 2. The initial selection of a location (e.g., *b*), in response to a scene presentation (e.g., *A*), is driven by the firing rates of the simulated cells selected by the presented scene (e.g., *C^Aa^, C^Ab^, C^Ac^, C^Ad^*). In the present case, neuron 2 has the highest firing rate initially, and tends to control the behavior more frequently early in the session. As the session progresses, the firing rate of the cell that corresponds to the correct location-scene pair increases (neuron 1), which increases the probability of making a correct response. With learning, the onset of *V*(*t*) steadily shifts toward the onset of the stimulus presentation in neurons 1, 3, and 4 (Fig. 3A,I,M) (Schultz et al., 1997; Suri and Schultz, 2001). Similarly, the offset of *V*(*t*) in neuron 2 shifts toward the onset of the stimulus presentation (Fig. 3E). These shifts are due to the learning rule in Eq. (4), which samples the information about the occurrence of a reward at the end of the trial, and, with increasing trial number, propagates it to earlier times within the trial.

The conditional intensity function of each neuron is computed using Eq. (6). Simulated spiking activity is generated as a doubly-stochastic Poisson process using the conditional intensity function. The raster plots of this activity reflect the temporal features of the underlying conditional intensity functions within and between trials (Fig. 3C,G,K,O). The average firing rate of neuron 1 in the delay period of the task increases with learning (Fig. 3D), whereas the firing rate of neuron 2 in the scene period decreases with learning (Fig. 3H). The firing rates of neurons 3 and 4 in the delay period exhibit a transient increase followed by decay toward the baseline (Fig. 3L,P).

### Simulated Neural Activity in Different Learning Quartiles

Figure 4 compares the activity of neuron 1 to the activity of a real changing cell in different quartiles of the session relative to the estimated learning trial of the network (from Fig. 3A of Wirth et al., 2003). The quartiles are determined using the methods of Wirth et al. (2003) as follows. The network's behavioral learning trial is estimated as explained in section “Behavioral learning trial estimate.” The session is divided into four quartiles relative to the behavioral learning trial such that the first two quartiles (Q1, Q2) correspond to the first and the second halves of the session before the learning trial, whereas the last two quartiles (Q3, Q4) correspond to the first and the second halves of the session after the behavioral learning trial. The firing rate of neuron 1 is estimated from its spiking activity using adaptive filtering as explained in Wirth et al. (2003), and the average activity per quartile is computed using this estimate. Figure 4 shows the results of this analysis for a real sustained changing cell and for neuron 1.

It is seen that the activity of the real changing cell is mostly within the 95% confidence interval of the estimated firing rate of neuron 1. These results are obtained using the same parameters as in Figure 3, without optimizing the parameters to fit the neural activity shown in Figure 4. This suggests that the within-trial time course of the learning-related change in the activity of this changing cell can be explained to a large extent by the temporal structure inherent in the reward-predictive value function.

### The Time Course of Expected Reward for Each Cell

**FIGURE 5:**
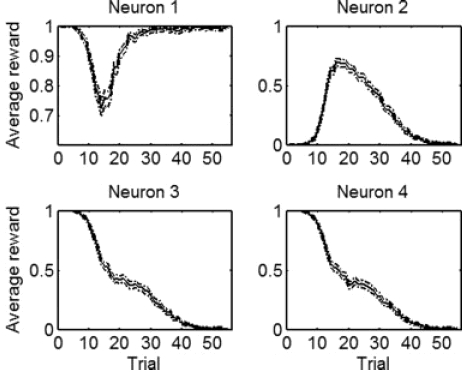
The expected reward of each cell during learning. The average reward received by each cell in 1,000 simulations. The dashed lines show the 95% binomial confidence intervals.

Figure 5 shows the average reward signal received by each neuron, which is computed using 1,000 simulated learning sessions. Initially the network's behavior is determined mostly by the activity of neuron 2. Because this is an incorrect neuron it does not receive reward when it succeeds in controlling behavior. As a result, its activity level decreases (Fig. 3H). Because of the reward inversion, the other cells receive positive reinforcement, which increases their activities (Fig. 3D,L,P). After a number of trials into the session, the neurons reach a comparable activity level. At this stage, each neuron is equally likely to win the competition. However, because the cells exhibit a medium activity level in their dynamic range relative to the parameters θ and τ, a winner cell's probability of selecting its associated location is around 0.5, leading to occasional incorrect choices when the winner is a correct cell, and to correct choices when the winner is an incorrect cell. This is indicated by the dip (peak) in the expected reward for neuron 1 (2), and the inflection point for the other neurons. Eventually, however, neuron 1 accumulates sufficient weight strength to take over the behavioral control, start to reliably select the correct location and receive positive reinforcement.

### The Network's Decision Making and Learning Trial

The network's probability of correct response, given the ensemble spiking activity, has the distribution shown in Figure 6A at each trial of a simulated learning session. The probability mass is initially collected near zero. This is because the network's response is initially dominated by the activity of neuron 2, which is an incorrect neuron. With learning, the probability mass shifts toward higher probabilities. Eventually, the 5th percentile of the distribution exceeds the chance level at trial 35, which is the network's learning trial in this session. The distribution is multimodal near the learning trial because the probability of correct response is small if the winner is an incorrect cell, and large otherwise. Figure 6B shows the behavioral learning curve of the network. For comparison, the learning curve estimated using the state-space model of learning (Wirth et al., 2003; Smith et al., 2004) is also plotted (Fig. 6C). The learning trial estimated by this method is IO(0.95) = 27. Figure 6D shows the distribution of the difference between the estimated and the actual learning trials in 1,000 simulations. The actual learning trial of the network is 38.8 ± 0.12 on the average (mean ± sem), whereas its estimated learning trial is 30.5 ± 0.17, which is significantly earlier (*P* = 0; paired, one-sided *t*-test). The probability of correct response at the actual learning trial is 0.95 ± 0.00024 (mean ± s.e.m. of the 999 simulations in Fig. 6D). The estimated probability of correct response at the IO(0.95) learning trial is 0.67 ± 0.0026 on the average (mean ± s.e.m. of the 963 simulations in Fig. 6D).

### Neural Change Trial of Neurons 1 and 2

Figure 7 shows the actual and the estimated neural change trials of neurons 1 and 2 during the simulated learning session shown in Figure 6, along with the distribution of the difference between the estimated and the actual neural change trials for each estimation method. The neural activity shows a consistent increase in panel A (*P* = 0.014) and decrease in panel D (*P* = 0), before the estimated learning trial, as in Figure 2 (see section “Slope of neural change before learning” for Methods). The activity significantly differs from the rate at the first trial at trial 22 and 17 for neurons 1 and 2, respectively. In 1,000 independent simulation sessions, the change point test (Siegel and Castellan, 1988) applied on the raw and the adaptive filtering estimates of the firing rate suggests that the neural change occurred at trials 26 ± 0.1 and 27.1 ± 0.0, respectively, for neuron 1, and at trials 17.5 ± 0.0 and 23.8 ± 0.1, for neuron 2 (mean ± sem), which are significantly later than the neural change trials defined here (*P* = 0, one-sided *t*-test). The difference is significantly larger for either neuron for the method that uses the adaptive filter estimate of the firing rate (*P* = 0; paired one-sided *t*-test).

## DISCUSSION

### Functional Explanation of Neural Activity

The present results suggest that the learning-related activity of the sustained-changing cells and the baseline-sustained changing cells identified by Wirth et al. (2003) may be explained within a common functional framework. Specifically, the sustained-changing cells that signal learning with an increased firing rate, and the baseline-sustained changing cells that signal learning with a decreased firing rate may be cells of the same type that differ in their state of mnemonic coding. For instance, the cell in Figure 2A may be learning to associate reward-predictive value with location-scene specific inputs using naïve synapses, whereas the cell in Figure 2B may be receiving inputs through channels that may have been previously paired with reward but are no longer reward-predictive in the present context. Such neurons may be activated by location-scene specific cues that may be common to multiple scene stimuli and may have been trained in a previous session but happen to be recorded in another session where the cues to which they respond are no longer reinforced. On the other hand, neurons 3 and 4 exhibit a transient activation pattern that resembles the hippocampal transient cells found in monkeys performing a conditional spatial response learning task (Cahusac et al., 1993) similar to the location-scene association task. These observations suggest that the present framework may provide a unified functional explanation for seemingly disparate findings on learning-related hippocampal neural activity.

An example of a sustained changing cell that signals learning with decreased firing rate is not available in Wirth et al. (2003). On the other hand, an example of a baseline-sustained changing cell that initially responds with decreased activity, and returns to the baseline level with learning is shown in different quartiles of a learning session in Figure 3B of Wirth et al. (2003). The activity of this cell also seems to exhibit the pattern of back propagating activity profile at the border of the scene and the delay periods of the task, although other aspects of its activity, such as the buildup and the decay of activity within the trial, are not readily explained by the present model. Also, it is not known whether the changing cells are pyramidal neurons or interneurons (Wirth et al., 2003). Identifying the cell type of the changing cells and the hippocampal substructures where different changing cell types are recorded from may shed more light into the functional role played by these cells during reinforcement learning. These issues may be addressed in future studies.

#### Significance of the reward inversion mechanism

The goodness-of-fit of the TD algorithm was significantly better when the algorithm received positive reinforcement at incorrect trials (Fig. 2A) or no reinforcement at correct trials (Fig. 2B) in fitting the activity of different changing cells. As a result of reward inversion, the activity of the model neurons changes before the model starts making correct responses. This may explain the significant trend of change that is observed in the firing rate of real changing cells before the subject starts making correct responses (Figs. 2 and 4), which suggests that information about the correct location-scene association starts being represented in the activity of these cells before it is detected in the monkey's behavior. It is proposed here that the changing cells may be receiving inverted reward signals if the reward signal is gated by competitive interactions among the cells such that the winners of the competition receive the actual reinforcement signal, whereas the losers receive an inverted reinforcement signal. For the reward inversion mechanism to be implemented in the brain, the activity of some neurons must correlate with the absence of reward. Neurons that show stronger task-related change in activity in unrewarded rather than rewarded trials have been observed in the dorsolateral prefrontal cortex, the orbitofrontal cortex, the striatum, and the pars reticulata of substantia nigra (Schultz, 2004) and may participate in the implementation of such a reward inversion mechanism.

#### Incorrect cells

Two types of incorrect cells were considered here. In one category (e.g., neuron 2), the cells signal a high reward-predictive value at the outset of a new session. In the other category (e.g., neurons 3 and 4), the cells do not signal a high reward-predictive value initially. As a consequence of the competition and the reward inversion mechanisms, the latter show an early increase in activity, followed by a decrease to baseline levels (Fig. 3L,P). These may therefore be referred to as transient incorrect cells. Cells that show such transient changes in learning-related activity were found in the hippocampus during the learning of a conditional spatial response task (transient cells, Cahusac et al., 1993), and in the supplementary eye field during the learning of conditional visuomotor associations (learning-selective cells, Chen and Wise, 1995). The activity of the transient cells was proposed to result from competitive interactions among hippocampal neurons (Cahusac et al., 1993).

The changing cells were identified on the basis of their significant correlation with the behavioral learning curve (Wirth et al., 2003). In the present results, the delay period activity of the transient incorrect cells passed the correlation test 50% of the time in 1,000 simulations. This relatively high rate of significant correlation was obtained partly because the activity of these cells did not fully return to the baseline activity level within the number of trials considered here. Also, the significance of their correlation with the behavioral learning curve depends on the model parameters. Here, the parameters were estimated using the firing rates of the cells shown in Figure 2. These cells show a very high correlation with the learning curve (0.96 and 0.83 as shown in Fig. 2A,C of Wirth et al., 2003). But the large majority of the significant correlations exhibited by the changing cells were smaller, as low as 0.1 (Fig. 2E of Wirth et al., 2003). If the model parameters were estimated from such changing cells, then the activity generated by the transient incorrect cells might have been too weak to be detected by the correlation test at any appreciable rate. These factors may explain why changing cells that fit the description of the transient incorrect cells were not reported by Wirth et al. (2003).

### Neural and Behavioral Change in the Model

Significant neural change occurs as early as the first trial in the network considered here. This is because the weights of at least one neuron always change after the first feedback signal is received in the form of a reward or lack thereof. From this point of view, the network's neural change trial is the first trial in the session. This situation is similar to the first synaptic change expressed by some of the neurons that participate in representing the association that is ultimately learned by the subject.

The change point test (Siegel and Castellan, 1988) applied on the raw and the adaptive filtering estimates of the firing rate suggested that the neural change occurred significantly later than the neural change trial defined here. This test assumes that the firing rate observations at consecutive trials form an ordered sequence, and that, initially, the distribution of the rates has one median, and, at some point there is a shift in the median of the distribution (Siegel and Castellan, 1988). It is important to note that this test reports the point where the evidence of change is strongest, rather than the earliest point of significant change. Therefore, significant changes may occur at trials earlier than the change trial reported by this method.

By contrast, the neural change trial defined here is the first trial at which the firing rate significantly differs from the rate at the first trial and remains so for the rest of the session. This explains why the change trial detected by this method was significantly earlier than the change trials detected by the application of the change point test on the raw or the filtered firing rates. This result suggests that the changing cells may have changed their activity before the estimated neural change points during the location-scene association task.

The behavioral learning trial of the network has been determined here using the distribution of the probability of making a correct response given the ensemble spiking activity of the model neurons. It was significantly later than the IO(0.95) learning trial. The discrepancy between these results is largely due to the differences between the definitions of the learning trial in these methods. Although the present analysis identifies the learning trial on the basis of the model's probability of generating an ensemble activity pattern that results in an abovechance probability of correct response with 95% confidence, the IO(0.95) learning trial is an estimator of the first trial at which the true probability of correct response exceeds the chance level and remains above that level for the rest of the session (see Appendix). As a result, the estimated probability of correct response may be arbitrarily close to the chance level at the IO(0.95) learning trial. For instance, it is 77% in Figures 2A,C of Wirth et al. (here Figs. 2A,B), and 39% in Figure 6C of Smith et al., 2004 (not shown here), which are different cases of the location-scene association task, where the chance level is 25%. This suggests that, in typical learning data, the IO(0.95) learning trial may be earlier than the learning trial determined using learning criteria that require a high probability of correct response, such as 90% correct, before accepting the occurrence of learning. These observations suggest that the learning trial could be defined in terms of a high percent correct criterion, such as 90% correct, that is the same for all cases of a given task. The insight provided by the network model suggests that, at such a learning trial, the neural activity under-lying the decision making process may give rise to an above-chance correct response probability with high confidence, as in Figure 6B (see Appendix).

These observations may guide the inference of when neural activity changes relative to behavior during learning. In Figures 2A and 2B, the neural activity exhibits a significant trend of change before the estimated learning trial, at trials where the subject makes incorrect responses, suggesting that information about the correct location-scene association is being expressed in the neural activity before the behavior changes. However, the estimated neural change trial (26 in A, 19 in B) occurs after the IO(0.95) learning trial (25 in A, 9 in B) in both of these figures. As explained above, the neural change trial estimation method that was used to obtain these estimates reports the trial where evidence of neural change is strongest, which may be several trials after the earliest trial of significant change. Also, these neural change trials were obtained using the adaptive filtering estimate of the firing rate, which yielded significantly late neural change trials when compared with those obtained from raw firing rate estimates in simulations. These observations suggest that the change in hippocampal neural activity leads behavior during the learning of the location-scene association task.

Although the network model generates its behavioral responses as a function of the firing rates of the neurons, this is not proposed to suggest that hippocampal neurons drive the motor behavior of a subject. Rather, the results suggest that the activity of certain hippocampal neurons may contain sufficient information about the reward-predictive value of different sets of task-relevant signals. The timing of this information suggests that it may be used for action selection. In this sense, such hippocampal neurons may be involved in driving the behavioral learning. Other brain areas that show learning-related activity correlated with reward predictive value may also play a role in this process (Liu and Richmond, 2000; Schultz, 2000; Barraclough et al., 2004; Seymour et al., 2004; Tanaka et al., 2004; Samejima et al., 2005; O'Doherty et al., 2006).

### Relation to Reward-Related Activity in Rodent and Primate Hippocampal Neurons

One of the leading theories of hippocampal function suggests that the primary function of the hippocampal system is to represent a cognitive map of space that serves spatial navigation (O'Keefe and Dostrovsky, 1971; O'Keefe and Nadel, 1978). Hippocampal place cell ensembles encode position-related information (Wilson and McNaughton, 1993; Brown et al., 1998; Okatan et al., 2005), which may form the basis of a cognitive map of space. They also encode correlates of nonspatial information experienced by a subject in different episodes within the same environment through changes in firing rate while maintaining position specificity (rate remapping; Leutgeb et al., 2005). Hippocampal place cells that change their firing rate at the same spatial location in a way that suggests that their activity is correlated with reward expectation (Hölscher et al., 2003) may be viewed as examples of rate remapping place cells. The present proposal that the activity of some hippocampal neurons may contain information about the rewardpredictive value of task-relevant signals is compatible with the notion of rate remapping. Given that nonspatial information may be corepresented with position information in hippocampal networks, the question arises as to how this information may contribute to the function of the hippocampus. In particular, is reward-related information, such as the reward-predictive value as defined within the temporal difference learning theory, corepresented with position information within the hippocampus, and if so, what does it contribute to hippocampal function? The results of some recent studies that found reward-related activity in the hippocampus may be interpreted in light of the present results to propose answers to these questions.

Several studies have recently provided evidence for activity related to reward and reward location in rat hippocampal place cells (Hölscher et al., 2003; Foster and Wilson, 2006; Lee et al., 2006; Ainge et al., 2007). Hölscher et al. (2003) recorded single CA1 neurons while rats explored an 8-arm maze and retrieved pellets at the end of each arm. They reported that, of the 31 hippocampal place cells that they identified, 11 showed enhanced firing activity when the animal entered a baited arm but did not fire when the arm was visited again after the bait had been retrieved. In another experiment, only four out of eight arms were baited. Of the 46 hippocampal neurons that were identified, which included cells that did or did not show place cell characteristics, all cells fired more in baited arms than in nonbaited ones. In a reversal task in which previously unbaited four arms were subsequently baited, Hölscher et al. observed an increase in neural activity in the newly baited arms. They interpreted these findings to suggest that the reward-dependent activity of some place cells may represent reward expectation. In these experiments, reward was either available or not on a maze arm. Further experiments that explore the dependence of such activity on graded modulations of expected reward may help determine whether the activity represents a reward expectation or a reward-predictive value.

**FIGURE 8:**
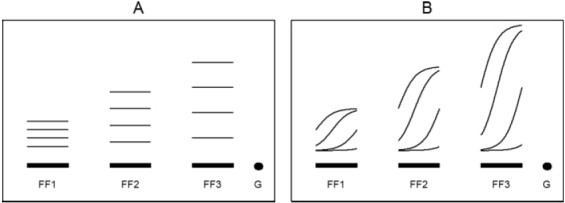
Predicted neural activity profiles during value learning. The graphs show the predicted time course of learning-related activity of three hypothetical hippocampal CA1 pyramidal neurons whose firing fields (FF), which may be temporal (episode field, Pastalkova et al., 2008) or spatial (place field), are located at different distances from the goal (G). The curves show the average predicted activity in four quartiles of a session at trials where the animal runs from the left to the right. The firing rates are assumed to increase with learning, as in Figure 4. The activities establish a value gradient that decays with increasing distance from the goal, as suggested by Foster and Wilson (2006). The learning related change in the activity of each cell may be isotropic within each firing field (A), or may start near the goal and propagate backward in time (B) as predicted by the model proposed here.

The proposal that the activity of some place cells may contain information about reward-predictive value is compatible with recent evidence suggesting that behavioral sequences are replayed in hippocampal place cells in reverse order during awake states (Foster and Wilson, 2006). Foster and Wilson showed that place cells that are sequentially activated as a rat runs back and forth on a linear track are reactivated in reverse order during brief pause intervals immediately following each lap while the rat consumed food reward from a food well. Foster and Wilson observed that this reverse replay of behavioral sequences coincided with hippocampal ripples and suggested that it might allow recently activated cells to be more strongly associated with a fast onset, slowly decaying dopamine signal to learn a representation of value as in TD learning models. They suggested that “this may provide a value gradient that the animal could follow during subsequent-goal finding behavior.” In this interpretation, the value gradient is represented over a population of cells such that the cells that are near the goal fire more than those that are far from the goal (Fig. 8). In other words, each cell learns the reward-predictive value of its place field, which increases as place cells approach the goal location. Foster and Wilson's explanation bridges the temporal gap between the activation of far place cells and the time when the reward is received using a model that suggests that place cells that are crossed during the trip toward the goal remain in a subthreshold excited state such that when the hippocampal ensemble is released from inhibition during ripples, the cells are reactivated in the order of increasing distance from the goal, allowing them to be paired with the slow decaying dopamine signal. This explanation is compatible with a reward-gated potentiation/depression of the recently-activated input streams of the place cells, such that when the animal crosses their place fields at a later time, the place cells may be driven through modified synapses and may signal the updated reward-predictive value. This interpretation may apply generally to hippocampal neurons, whether they are driven mainly by position signals or other types of input. In this way, the reverse replay phenomenon may represent the implementation of a general learning rule in a polymodal memory space. Such a reward-modulated synaptic update would result in the backward propagation of reward-predictive value across a population of cells with increasing distance from the goal location. If the reward-predictive value were acquired according to a mechanism similar to the model proposed here, then a similar value gradient would also be observed within the temporal spiking activity patterns of individual cells, such that the activity would change with learning first at the place field border nearest the goal location, and then would propagate backward in time toward the distal end of the place field as learning progressed (Fig. 8B).

The place field plasticity observed by Ainge et al. (2007) in a recent study is compatible with this time course. Ainge et al. (2007) provided evidence that rat CA1 place cells encode intended destination on a maze with multiple choice points. During the learning of a new set of two randomly selected rewarded destinations out of four endpoints on the maze, they observed an interesting pattern of place field plasticity in cells that changed their firing dynamically within a session. Most (37/46) of these cells were silent at the beginning of a session and then developed robust place fields after a number of trials. The place fields of such cells started near a choice point or a goal location, and appeared to expand backwards toward the start box (Fig. 6A of Ainge et al., 2007). Backward field expansion on linear tracks has been previously reported and interpreted to reflect Hebbian synaptic plasticity between sequentially activated place cells (Mehta et al., 1997). However Ainge et al. (2007) pointed out that it was not clear how this type of mechanism could account for the goal sensitive activity they observed, “because the goal encoding seemed to be in the form of large differences of firing rate in the same location (i.e., rate remapping) and not a shift in field locations between trajectories.”

Recent evidence suggests that different cell assembly sequences are internally generated in the hippocampal area CA1 and these sequences predict the future choice of a rat in selecting alternating trajectories on a maze (Pastalkova et al., 2008). This suggests that different cell assemblies may have been activated in the hippocampus during trips to different goals in the experiment of Ainge et al. (2007). Then, the goal sensitive backward expansion of the place fields might be explained as a function of both a goal-dependent activation of cell assemblies, and Hebbian synaptic plasticity between sequentially activated place cells in that cell assembly. Alternatively, the co-occurrence of goal-dependent rate remapping and the backward expansion of place fields may be compared with the backward propagation of the reward-predictive value in the correct cells of the present model. If the within-trial time during the delay period in Figure 4 is viewed as the time spent during the trip from the start box to the goal location, it would be seen that the increased activity of a neuron that signals reward-predictive value would first emerge near the goal location, and then would propagate backwards toward the start box (backward expansion), and this would occur only during trips to the correct goal location (goal-dependent rate remapping). The present model suggests that such activity would be observed if these cells signal the reward-predictive value of an input signal that is available before the goal is reached. These may be goal-specific input signals that are available to the animal during its trip toward the goal. One such signal is the neural representation of the goal itself (Montague et al., 2004).

If some hippocampal neurons are driven by the neural representations of a subject's goals, this could also explain the activity that emerges before and terminates at the choice points. Such activity could be interpreted as the reward predictive value of the representation of the rat's planned action at that choice point. The representation of such subgoals may be turned off after the choice point is passed, turning off the drive to the hippocampal neurons that may be representing the reward-predictive value of such subgoals, which would cause the associated place fields to terminate at the choice point. The notion of a sequence of subgoals leading to a goal is reminiscent of sequentially activated goal dependent cell assemblies found by Pastalkova et al. (2008) in hippocampal area CA1. Given that recently activated CA1 pyramidal neurons are reactivated at the goal in reverse order in a way that may pair them with a fast onset, slowly decaying dopamine signal to learn a representation of value (Foster and Wilson, 2006), it is conceivable that reward-predictive value may be propagated backward in time across cells that make up an assembly (Fig. 8).

In addition to the backward field expansion phenomenon, Ainge et al. (2007) also observed place fields that translocated from the goal location to the start location. Translocation of place fields has also been observed in a T-maze alternation task, although in the forward direction toward a goal location (Lee et al., 2006). Such translocation never continued beyond the goal location. Between consecutive laps of the T-maze alternation task, the processes of neural plasticity that underlie the forward translocation of the place fields may occur during either the theta state (Buzsaki, 2002) or during the ripple episodes within the large irregular activity states (Foster et al., 1989), or both (Lee et al., 2006). One of the explanations that Lee et al. (2006) provided for the forward translocation of place fields suggested that the activity of these cells might be influenced by a value gradient that increases toward the goal location, consistent with the findings of Foster and Wilson (2006). In combination with the present results, this explanation suggests that hippocampal networks may form goal and context sensitive representations that are rapidly shaped and modified during learning, and that information about value gradients may explain different aspects of learning-related hippocampal neural activity.

Reward-place dependent activity in hippocampal neurons has also been observed in primates (reward-place cells, Rolls and Xiang, 2005). Rolls and Xiang recorded neural activity from the hippocampus of a rhesus macaque performing a reward-place association memory task in which the subject had to associate different locations in an entire visual scene with different rewards. Rolls and Xiang found that the activity level of some neurons depended on both the part of a visual scene that was viewed by the animal and the type of reward (more-preferred or less-preferred) that was available when that part of the scene was touched. Thus, Rolls and Xiang found evidence that the activity of some hippocampal neurons represents reward associations of places viewed by the animal, and suggested that the concept that the primate hippocampus is involved in object-place event memory might be extended to remembering goals available at different spatial locations.

In the experiments of Rolls and Xiang (2005), the subjects fixated to the target visual areas in less than 150 ms, the neurons in question had typical latencies of 200 ms (Fig. 3 of Rolls and Xiang, 2005), and the subject's touch response latency was typically between 500 and 1,000 ms. Thus, the neurons were activated after the subject fixated on the target areas and maintained fixation during the reach. This suggests that the cells might be driven by the spatial view cells (Rolls, 1999; Rolls and Xiang, 2005). Unlike the experiments of Rolls and Xiang (2005), in Wirth et al. (2003), the subjects maintained fixation throughout the scene and the delay periods before making an eye movement response, which kept the spatial view constant during these periods. Yet, the activity of the changing cells was selective to location-scene pairs, where different target locations subtended different viewing angles and presumably would activate view cell populations other than those activated while fixating. Unless certain view cells are activated by covert attention processes when the subject is not overtly viewing the associated “space out there,” the response of the changing cells may not be assumed to be driven by input from the view cells associated with the target locations. In other words, the changing cells may be functionally different from the reward-place cells identified by Rolls and Xiang (2005). Because the changing cell response develops within the trial while the subject maintains fixation, it is possible that it is driven by the representation of the goal of making the impending eye movement to a particular location. Neurons that signal the location of such impending eye movements have been identified in the dorsolateral prefrontal cortex in macaque monkeys performing a delayed-response working memory task (Funahashi et al., 1989) that shares similar event sequence and behavioral demands as the location-scene association task, and they may interact with hippocampal neurons through dual pathways connecting these structures (Goldman-Rakic et al., 1984, Goldman-Rakic, 1987).

Reward is arguably one of the most important aspects of an agent's episodic experience of what happens where and when. Given its crucial role in encoding and recalling episodic memories (Eichenbaum et al., 1999; Eichenbaum and Fortin, 2005), information about what contextual signals reliably predict reward, and how much reward they predict, may be integrated by the hippocampus to the representation of the agent's ongoing episodic experience (Rolls and Xiang, 2005). Such reward-related information would be crucial to the formulation of a declarative or explicit representation of task rules in reinforcement learning experiments. The hippocampus is crucial for declarative memory in humans (Squire et al., 1993; Schacter and Tulving, 1994), and is thought to mediate declarative-like memory representations in animals (Eichenbaum, 1999). Recent evidence suggests that human patients with basal ganglia lesions, including bilateral lesions to the striatum, may rely on a hippocampus-based declarative learning strategy in associative reward-based learning (Bellebaum et al., 2008). It is possible that hippocampal neural activity that contains reward-related information may constitute the reward-related aspects of a declarative or explicit relational memory representation of task contingencies. The present results make specific predictions regarding the time course with which such reward-related hippocampal neural signals would change with changing reward schedules. These predictions may be tested in future experiments in order to decisively determine the relationship between hippocampal neural activity and reward-predictive value.

## Model's Predictions and Future Studies

### Experimental studies

Interpreting the results of Hölscher et al. (2003), Rolls and Xiang (2005), Ainge et al. (2007), and Pastalkova et al. (2008) in view of the present results leads to predictions concerning the time course of the learning-related change in the activity of some of the hippocampal neurons identified in these studies. After learning occurs and neural activity asymptotes during a session in the experiments of Wirth et al. (2003), Ainge et al. (2007), and Pastalkova et al. (2008), changing the amount of the reward delivered to the subject in further trials may result in a change in neural activity, as in the experiments of Hölscher et al. (2003) and Rolls and Xiang (2005). The time course of this change may follow the time course predicted by the *V*(*t*) signal of the TD algorithm. More generally, analyzing the activity of the cells reported by Hölscher et al. (2003), Rolls and Xiang (2005), Ainge et al. (2007), and Pastalkova et al. (2008) during the act of learning may reveal that these cells exhibit the characteristic backward spread of the *V*(*t*) signal from the time of reward delivery toward the time of stimulus onset and that this activity is modulated by the amount of reward that the subject learns to expect. When analyzed in different learning quartiles, such activity may exhibit the activity profile suggested in Figure 8B.

### Computational studies

Future studies that use the present framework to jointly explain neural ensemble spiking activity and binary behavioral responses may use likelihood-based model selection methods (Burnham and Anderson, 2002) to further explore the mechanisms by which learning-related ensemble neural spiking activity gives rise to adaptive decision making in biological nervous systems.

Alternative competitive learning models that explain the present data may be explored in future studies. Importantly, competitive learning by itself does not explain how neural activity in a reinforcement-learning system would change before the system's behavior changes. The present article identifies this as a computational problem and proposes the reward-inversion mechanism as a plausible solution, which may be further explored in future modeling studies.

Future studies may generalize the present framework by replacing each model cell by populations, and by modeling the interactions among all neurons using point process network likelihood models (Okatan et al., 2005; see Appendix). The link between neural activity and behavior may also be formulated to allow action selection to be performed by ensembles, using rules such as the softmax action selection (Sutton and Barto, 1998), or simple extensions of Eq. (8) within the GLM framework such as


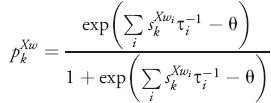


where *w* now represents a neural ensemble of winner neurons that may be defined according to a criterion.

### Neurobiological Models

Algorithmically, *V*(*t*) acts as a memory of the expected future reward, which is acquired through learning. Its temporal difference is used to compute the TD error signal δ(*t*). Neurobiological models of the TD algorithm propose that δ(*t*) corresponds to the output of the midbrain dopamine neurons, which may receive the temporal difference of *V*(*t*) through a circuit involving the limbic striatum (Houk et al., 1995; Schultz, 1998; Yin and Knowlton, 2006). The limbic striatum in turn receives inputs from a variety of areas, including the limbic cortex and the hippocampus (Kunishio et al., 1996; Friedman et al., 2002; Jung and Hong, 2003). The activity of the striatal neurons is influenced by reward several seconds before its occurrence, suggesting that they receive information regarding an upcoming reward from a predictive memory signal (Apicella et al., 1992). The activity of these neurons was modeled in a previous study using *V*(*t*) (Suri and Schultz, 2001). The present results are compatible with the possibility that some hippocampal neurons may be involved in the representation of the predictive memory signal that is thought to drive the striatal neurons.

Sutton and Barto, the pioneers of the TD learning algorithm, indicate that “almost all reinforcement learning algorithms are based on estimating value functions—functions of states (or of state-action pairs) that estimate how good it is for the agent to be in a given state (or how good it is to perform a given action in a given state)” (Sutton and Barto, 1998, [p.68]). The activity of the changing cells exhibits key characteristics of a predictive value function that estimates how rewarding it will be for the animal to choose a particular location given a particular scene (Figs. 2 and 4). Because the activity of each changing cell seems to be specific to a single location- scene pair (Wirth et al., 2003), information about the value function associated with the overall task may be represented across a network of these cells. Future experiments that probe the relation between hippocampal neural activity and reward predictive value in view of the present findings may further explore such representations.

## Figures and Tables

**FIGURE 9:**
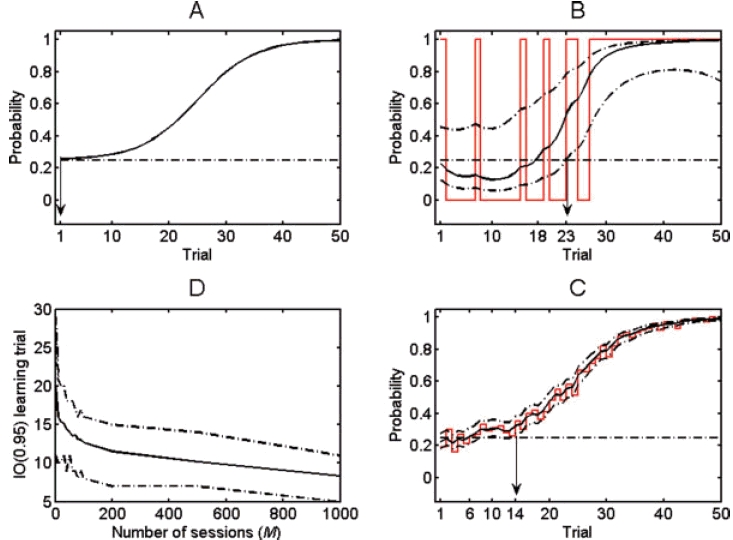
The IO(0.95) learning trial. (A) The probability of correct response as a function of trial number for a hypothetical learning system. Dashed line: chance level (0.25). The probability of correct response is greater than chance at all trials. The IO(0.95) learning trial is an estimator of the first trial at which the probability of correct response exceeds the chance level, which occurs here at trial 1 (arrow). (B, C) The state-space model of learning (Wirth et al., 2003; Smith et al., 2004) is used to compute the IO(0.95) learning trial using behavioral data generated in *M* = 1 (B) or *M* = 100 (C) independent simulated learning sessions using the learning curve in (A). Red curves: the proportion of correct responses at each trial. Solid black line: the learning curve estimated using the state-space model. Dashed lines: the 90% confidence interval of this estimate. The IO(0.95) learning trial is indicated by the arrow. (D) The plot shows the mean (solid line) and the 95% confidence interval (dashed lines) of the IO(0.95) learning trial as a function of the number of sessions, *M*. The confidence interval is constructed using 100 independent estimates of the IO(0.95) learning trial at each *M*.

## APPENDIX: THE JOINT PROBABILITY DENSITY MODEL

Let *D^Xy^* denote a neuron that is assumed to represent the cell *C^Xy^* of the model, and let  be the sample path of the counting process associated with the spike train of neuron *D^Xy^*, observed during the time interval [0,*T*] at interleaved trial *q*. The sample path is a right continuous function that jumps one at the spike times and is constant otherwise (Snyder and Miller, 1991). In this way,  counts the number and location of the spikes of neuron *D^Xy^* at trial *q*. Let *Z_q_* ∈ {*A,B,C,D*} denote the scene that is presented at trial *q, z_q_* ∈ {*a,b,c,d*} denote the associated correct location, and *n_q_* denote the outcome of the subject's response at that trial, such that *n_q_* is 1 if the response is correct and 0 if it is incorrect. Then the joint probability density of the spikes fired by the neuron *D^Zqy^* at trial *q* is (Daley and Vere-Jones, 2003)

(A1)

where,  is the activity history of the four *Z_q_*-responsive neurons during the time interval [0,*T*] at interleaved trials 1 to *q – 1*, and  is given by Eq. (6). The value function  (*t*) is computed iteratively, using Eqs. (2)–(4), from the initial weights and the data available at trials 1 to *q* – 1.

The probability *p_q_* of making a correct response at trial *q* is computed by the model using Eqs. (7) and (8). In Eq. (7), the variable *y* is set to *z_q_*. In Eq. (8), the spike count  is , where the index *w* of the winner neuron is , and [*t_i_*, *t_f_*] is a task period. Then, the probability mass function of *n_q_* is

(A2)

Assuming that the neurons activated by the scene *z_q_* are conditionally independent in the interval [0,*T*] given the neural and behavioral data at trials 1 to *q* – 1, the joint probability density of the spiking activity of all neurons and the behavioral responses at all trials across the entire session is

(A3)

where, *Q* is the total number of interleaved trials, *Z* is the vector of all presented scenes, and *z* is the vector of associated correct locations. Viewed as a function of the model parameters given the data, Eq. (A3) is the likelihood function of the model's parameter vector. Thus, the maximum likelihood estimate of the parameter vector can be computed by maximizing Eq. (A3) with respect to the parameters.

In Eq. (A3), the competition between the neurons may be implemented through lateral inhibition using a history-dependent conditional intensity function for each neuron, such that the spiking activity of each neuron depends on the recent activity history of itself and of its competitors (Chornoboy et al., 1988; Okatan et al., 2005). The joint probability density of neural and behavioral data under this implementation is again given by Eq. (A3), in which the joint probability density of the spikes fired by the neuron *D^Zqy^* at trial *q* is

(A4)

where  is the ensemble spiking history of the *z_q_*-responsive neurons in the interval [*t – W, t*) with *W* specifying how far the history dependence extends into the past (Okatan et al., 2005).

### The Dynamic Range of the Weights and the Value Function in the Learning Task

In the learning task considered here the reward is all or none (1 or 0) and is only available at the end of the trial. The weights and the value function vary between 0 and 1 under these conditions if α, γ, and the initial weights are between 0 and 1.

### Proof

The inputs to the simulated changing cells are a series of time-delayed pulses (Montague et al., 1996; Schultz et al., 1997; Suri and Schultz, 2001; O'Doherty et al., 2003).

(A5)

The reward satisfies *r_k_*(*t*) ∈ [0,1] for all *k* and *t*. From Eqs. (2) and (A5), , and from Eqs. (3) and (4)

(A6)

After the model receives feedback (reward, or lack thereof) at time *T*, the predicted value is zero at *t* = *T* + 1, that is, *wk*^*T*+1^ = 0 for all *k*. For *t* = *T*, denoting *wk^T^* by *y*[*k*] and *rk*(*T*) by *z*[*k*], Eq. (A6) gives

(A7)

With , and 0 ≤ α ≤ 1, for *k* > 1 Eq. (A7) yields

(A8)

implying that  for all *k*.

For *t* < *T*, *r_k_*(*t*) = 0 for all *k*. For *t* = *T* – 1, denoting  by *y*[*k*],  by *z*[*k*], and with , and 0 ≤ γ ≤ 1, Eq. (A6) turns into Eq. (A7), which again yields Eq. (A8), implying that  for all *k*. The in equality  is proven by induction for all *t* and *k*.

### The IO(0.95) Learning Trial

The IO(0.95) learning trial is an estimator of the first trial at which the true learning curve exceeds the chance level and remains above that level for the rest of the session. To show this, simulated binary response data are generated for *K* = 50 trials in a variable number *M* of independent sessions using a known *p* that is related to the trial number *k* according to

(A9)

with *p*_0_ = 0.25, *p_f_* =; 1, δ = 25, γ = 0.2 (Fig. 9A). This procedure generates behavioral responses *n_m,k_*, where 1 ≤ *m* ≤ *M* is the session number. The curve in Figure 9A is one of the curves used by Smith et al. (2004) to compare the performance of different methods in estimating the learning trial. According to these parameters, the chance level is *p*_0_ = 0.25, or 25% correct.

Figure 9 shows the system's learning curve estimated using the state-space model of learning for *M* = 1 (panel B) and *M* = 100 (panel C). The IO(0.95) learning trial is the first trial at which the lower bound of the 95% confidence interval of the estimated learning curve exceeds the chance level and remains above that level for the rest of the session. Note that the confidence interval is narrower for *M* = 100, and, as a result, the IO(0.95) learning trial occurs earlier in the trial for *M* = 100 (trial 14) vs. *M* = 1 (trial 23).

Figure 9D shows this trend for a range of *M* between 1 and 1,000. The IO(0.95) learning trial decreases as *M* increases, indicating that learning is accepted to occur at progressively earlier trials as more information is accumulated about the performance of the system. In theory, this decrease continues until the IO(0.95) learning trial reaches trial 1 in this example, because, according to Eq. (A9), the true probability of correct response is 0.2561 at trial 1, which is above the chance level of 0.25 (Fig. 9A).

### Learning trial based on the estimated learning curve

Most studies use a high percentage of correct performance, such as 80–90% throughout a session, or in a moving block of 10–20 trials, as a learning criterion (Eichenbaum et al., 1986; Sappington and Goldman, 1994; Gheusi et al., 1997; Knepper and Kurylo, 1998; Chapman et al., 1999; Schoenbaum et al., 1999; Bellebaum et al., 2008; Cacucci et al., 2008). This approach may also be used with the state-space model of learning, in analyses where this model may be used to estimate the learning curve. For instance, the learning trial may be defined as the first trial at which the estimated learning curve exceeds the 95% level and stays above this level for the rest of the session (e.g., trial 35 in Fig. 6C). Indeed, the true probability of correct response of the network was 0.94 ± 0.0023 (mean ± s.e.m.) at this learning trial in 953 simulated learning sessions, suggesting that this learning trial may be used in practice.

## References

[b1] Ainge JA, Tamosiunaite M, Woergoetter F, Dudchenko PA (2007). Hippocampal CA1 place cells encode intended destination on a maze with multiple choice points. J Neurosci.

[b2] Apicella P, Scamati E, Ljungberg T, Schultz W (1992). Neuronal activity in monkey striatum related to the expectation of predictable environmental events. J Neurophysiol.

[b3] Attwell D, Iadecola C (2002). The neural basis of functional brain imaging signals. Trends Neurosci.

[b4] Barraclough DJ, Conroy ML, Lee D (2004). Prefrontal cortex and decision making in a mixed-strategy game. Nat Neurosci.

[b5] Barto AG, Sutton RS (1982). Simulation of anticipatory responses in classical conditioning by a neuron-like adaptive element. Behav Brain Res.

[b6] Barto AG, Sutton RS, Anderson CW (1983). Neuronlike adaptive elements that can solve difficult learning problems. IEEE Trans Syst Man Cybern.

[b7] Bellebaum C, Koch B, Schwarz M, Daum I (2008). Focal basal ganglia lesions are associated with impairments in reward-based reversal learning. Brain.

[b8] Brown EN, Frank LM, Tang D, Quirk M, Wilson MA (1998). A statistical paradigm for neural spike train decoding applied to position prediction from ensemble firing patterns of rat hippocampal place cells. J Neurosci.

[b9] Brown EN, Nguyen DP, Frank LM, Wilson MA, Solo V (2001). An analysis of neural receptive field plasticity by point process adaptive filtering. PNAS.

[b10] Burnham KP, Anderson DR (2002). Model Selection and Multimodel Inference.

[b11] Buzsaki G (2002). Theta oscillations in the hippocampus. Neuron.

[b12] Cacucci F, Yi M, Wills TJ, Chapman P, O'Keefe J (2008). Place cell firing correlates with memory deficits and amyloid plaque burden in Tg2576 Alzheimer mouse model. PNAS.

[b13] Cahusac PM, Rolls ET, Miyashita Y, Niki H (1993). Modification of the responses of hippocampal neurons in the monkey during the learning of a conditional spatial response task. Hippocampus.

[b14] Chapman PF, White GL, Jones MW, Cooper-Blacketer D, Marshall VJ, Irizarry M, Younkin L, Good MA, Bliss TVP, Hyman BT, Younkin SG, Hsiao KK (1999). Impaired synaptic plasticity and learning in aged amyloid precursor protein transgenic mice. Nat Neurosci.

[b15] Chen LL, Wise SP (1995). Neuronal activity in the supplementary eye field during acquisition of conditional oculomotor associations. J Neurophysiol.

[b16] Chornoboy ES, Schramm LP, Karr AF (1988). Maximum likelihood identification of neuronal point process systems. Biol Cybern.

[b17] Daley D, Vere-Jones D (2003). An Introduction to the Theory of Point Processes.

[b18] Dempster AP, Laird NM, Rubin DB (1977). Maximum likelihood from incomplete data via the EM algorithm. J R Stat Soc B.

[b19] Efron B, Tibshirani RJ (1998). An Introduction to the Bootstrap.

[b20] Eichenbaum H (1999). The hippocampus and mechanisms of declarative memory. Behav Brain Res.

[b21] Eichenbaum H, Fortin NJ (2005). Bridging the gap between brain and behavior: Cognitive and neural mechanisms of episodic memory. J Exp Anal Behav.

[b22] Eichenbaum H, Fagan A, Cohen NJ (1986). Normal olfactory discrimination learning set and facilitation of reversal learning after medial-temporal damage in rats: Implications for an account of preserved learning abilities in amnesia. J Neurosci.

[b23] Eichenbaum H, Dudchenko P, Wood E, Shapiro M, Tanila H (1999). The hippocampus, memory, and place cells: is it spatial memory or a memory space?. Neuron.

[b24] Foster DJ, Wilson MA (2006). Reverse replay of behavioural sequences in hippocampal place cells during the awake state. Nature.

[b25] Foster TC, Castro CA, McNaughton BL (1989). Spatial selectivity of rat hippocampal neurons: Dependence on preparedness for movement. Science.

[b26] Friedman DP, Aggleton JP, Saunders RC (2002). Comparison of hippocampal, amygdala, and perirhinal projections to the nucleus accumbens: Combined anterograde and retrograde tracing study in the macaque brain. J Comp Neurol.

[b27] Funahashi S, Bruce CJ, Goldman-Rakic P (1989). Mnemonic coding of visual space in the monkey's dorsolateral prefrontal cortex. J Neurophysiol.

[b28] Gheusi G, Goodall G, Dantzer R (1997). Individually distinctive odours represent individual conspecifics in rats. Anim Behav.

[b29] Goldman-Rakic PS, Mountcastle VB, Plum F, Geiger SR (1987). Circuitry of primate prefrontal cortex and regulation of behavior by representational memory. The Nervous System.

[b30] Goldman-Rakic PS, Selemon LD, Schwartz ML (1984). Dual pathways connecting the dorsolateral prefrontal cortex with the hippocampal formation and parahippocampal cortex in the rhesus monkey. Neuroscience.

[b31] Haykin S (1996). Adaptive Filter Theory.

[b32] Houk JC, Adams JL, Barto AG, Houk JC, Davis JL, Beiser DG (1995). A model of how the basal ganglia generate and use reward signals that predict reinforcement. Models of Information Processing in the Basal Ganglia.

[b33] Hölscher C, Jacob W, Mallot HA (2003). Reward modulates neuronal activity in the hippocampus of the rat. Behav Brain Res.

[b34] Jung Y, Hong S (2003). Organization of projections from the medial temporal cortical areas to the ventral striatum in macaque monkeys. Korean J Biol Sci.

[b35] Kaelbling LP, Littman ML, Moore AW (1996). Reinforcement learning: A survey. J Artif Intell Res.

[b36] Kakade S, Dayan P (2000). Dopamine bonuses. NIPS.

[b37] Knepper BR, Kurylo DD (1998). Effects of nitric oxide synthase inhibitor N^G^-Nitro-l-Arginine Methyl Ester on spatial and cued leaning. Neuroscience.

[b38] Kunishio K, Ohmoto T, Haber SN (1996). Topographic organization of the ventral striatum afferent projection from amygdaloid complex and hippocampal formation. No To Shinkei.

[b39] Lee I, Griffin AL, Zilli EA, Eichenbaum E, Hasselmo ME (2006). Gradual translocation of spatial correlates of neuronal firing in the hippocampus toward prospective reward locations. Neuron.

[b40] Leutgeb S, Leutgeb JK, Barnes CA, Moser EI, McNaughton BL, Moser MB (2005). Independent codes for spatial and episodic memory in hippocampal neuronal ensembles. Science.

[b41] Liu Z, Richmond BJ (2000). Response differences in monkey TE and perirhinal cortex: Stimulus association related to reward schedules. J Neurophysiol.

[b42] Ljungberg T, Apicella P, Schultz W (1992). Responses of monkey dopamine neurons during learning of behavioral reactions. J Neurophysiol.

[b43] McClure SM, Berns GS, Montague PR (2003). Temporal prediction errors in a passive learning task activate human striatum. Neuron.

[b44] McClure SM, York MK, Montague PR (2004). The neural substrates of reward processing in humans: The modern role of fMRI. Neuroscientist.

[b45] McCullagh P, Nelder JA (1989). Generalized Linear Models.

[b46] Mehta MR, Barnes CA, McNaughton BL (1997). Experience-dependent, asymmetric expansion of hippocampal place fields. PNAS.

[b47] Montague PR, Dayan P, Person C, Sejnowski TJ (1995). Bee foraging in uncertain environments using predictive hebbian learning. Nature.

[b48] Montague PR, Dayan P, Sejnowski TJ (1996). A framework for mesencephalic dopamine systems based on predictive Hebbian learning. J Neurosci.

[b49] Montague PR, Hyman SE, Cohen JD (2004). Computational roles for dopamine in behavioral control. Nature.

[b50] Montague PR, King-Casas B, Cohen JD (2006). Imaging valuation models in human choice. Annu Rev Neurosci.

[b51] Niv Y, Duff MO, Dayan P (2005). Dopamine, uncertainty and TD learning. Behav Brain Funct.

[b52] O'Doherty JP, Dayan P, Friston K, Critchley H, Dolan RJ (2003). Temporal difference models and reward-related learning in the human brain. Neuron.

[b53] O'Doherty JP, Buchanan TW, Seymour B, Dolan RJ (2006). Predictive neural coding of reward preference involves dissociable responses in human ventral midbrain and ventral striatum. Neuron.

[b54] Okatan M, Wilson MA, Brown EN (2005). Analyzing functional connectivity using a network likelihood model of ensemble neural spiking activity. Neural Comput.

[b55] O'Keefe J, Dostrovsky J (1971). The hippocampus as a spatial map. Preliminary evidence from unit activity in the freely-moving rat. Brain Res.

[b56] O'Keefe J, Nadel L (1978). The Hippocampus as a Cognitive Map.

[b57] Pastalkova E, Itskov V, Amarasingham A, Buzsáki G (2008). Internally generated cell assembly sequences in the rat hippocampus. Science.

[b58] Rolls ET (1999). Spatial view cells and the representation of place in the primate hippocampus. Hippocampus.

[b59] Rolls ET, Xiang JZ (2005). Reward-spatial view representations and learning in the primate hippocampus. J Neurosci.

[b60] Romo R, Schultz W (1990). Dopamine neurons of the monkey midbraContingencies of responses to active touch during self-initiated arm movements. J Neurophysiol.

[b61] Samejima K, Ueda Y, Doya K, Kimura M (2005). Representation of action-specific reward values in the striatum. Science.

[b62] Sappington BF, Goldman L (1994). Discrimination learning and concept formation in the Arabian horse. J Anim Sci.

[b63] Schacter DL, Tulving E, Schacter DL, Tulving E (1994). What are the memory systems of 1994?. Memory Systems.

[b64] Schoenbaum G, Chiba AA, Gallagher M (1999). Neural encoding in orbitofrontal cortex and basolateral amygdala during olfactory discrimination learning. J Neurosci.

[b65] Schultz W (1998). Predictive reward signal of dopamine neurons. J Neurophysiol.

[b66] Schultz W (2000). Multiple reward signals in the brain. Nat Rev Neurosci.

[b67] Schultz W (2004). Neural coding of basic reward terms of animal learning theory, game theory, microeconomics and behavioural ecology. Curr Opin Neurobiol.

[b68] Schultz W, Apicella P, Ljungberg T (1993). Responses of monkey dopamine neurons to reward and conditioned stimuli during successive steps of learning a delayed response task. J Neurosci.

[b69] Schultz W, Dayan P, Montague RR (1997). A neural substrate of prediction and reward. Science.

[b70] Seymour B, O'Doherty JP, Dayan P, Koltzenburg M, Jones AK, Dolan RJ, Friston KJ, Frackowiak RS (2004). Temporal difference models describe higher-order learning in humans. Nature.

[b71] Siegel S, Castellan NJ (1988). Nonparametric Statistics for the Behavioral Sciences.

[b72] Singh S, Bertsekas D (1997). Reinforcement learning for dynamic channel allocation in cellular telephone systems.

[b73] Smith AC, Brown EN (2003). State-space estimation from point process observations. Neural Comput.

[b74] Smith AC, Frank LM, Wirth S, Yanike M, Hu D, Kubota Y, Graybiel AM, Suzuki WA, Brown EN (2004). Dynamic analysis of learning in behavioral experiments. J Neurosci.

[b75] Snyder D, Miller M (1991). Random Point Processes in Time and Space.

[b76] Squire LR, Knowlton B, Musen G (1993). The structure and organization of memory. Annu Rev Psychol.

[b77] Suri RE, Schultz W (1999). A neural network model with dopamine-like reinforcement signal that learns a spatial delayed response task. Neuroscience.

[b78] Suri RE, Schultz W (2001). Temporal difference model reproduces anticipatory neural activity. Neural Comput.

[b79] Sutton RS (1988). Learning to predict by the method of temporal difference. Machine Learn.

[b80] Sutton RS, Barto AG (1981). Toward a modern theory of adaptive networks: Expectation and prediction. Psychol Rev.

[b81] Sutton RS, Barto AG (1987). A temporal-difference model of classical conditioning.

[b82] Sutton RS, Barto AG (1998). Reinforcement Learning: An Introduction.

[b83] Tanaka SC, Doya K, Okada G, Ueda K, Okamoto Y, Yamawaki S (2004). Prediction of immediate and future rewards differentially recruits cortico-basal ganglia loops. Nat Neurosci.

[b84] Tesauro G (1994). TD-Gammon, a self-teaching backgammon program, achieves master-level play. Neural Comput.

[b85] Waelti P, Dickinson A, Schultz W (2001). Dopamine responses comply with basic assumptions of formal learning theory. Nature.

[b86] Wilson MA, McNaughton BL (1993). Dynamics of the hippocampal ensemble code for space. Science.

[b87] Wirth S, Yanike M, Frank LM, Smith AC, Brown EN, Suzuki WA (2003). Single neurons in the monkey hippocampus and learning of new associations. Science.

[b88] Yin HH, Knowlton BJ (2006). The role of the basal ganglia in habit formation. Nat Rev Neurosci.

